# BMP3 expression by osteoblast lineage cells is regulated by canonical Wnt signaling

**DOI:** 10.1002/2211-5463.12347

**Published:** 2017-12-16

**Authors:** Shoichiro Kokabu, Vicki Rosen

**Affiliations:** ^1^ Department of Developmental Biology Harvard School of Dental Medicine Boston MA USA; ^2^ Division of Molecular Signaling and Biochemistry Department of Health Promotion Kyushu Dental University Kitakyushu Japan; ^3^ Department of Oral and Maxillofacial Surgery Faculty of Medicine Saitama Medical University Moroyama‐machi Iruma‐gun Japan

**Keywords:** osteoblast, osteoblastogenesis, osteocyte, osteopenia, osteoporosis, regenerative medicine

## Abstract

Bone morphogenetic protein (BMP) and canonical Wnt (cWnt) signaling factors are both known to regulate bone mass, fracture risk, fracture repair, and osteoblastogenesis. BMP3 is the most abundant BMP and negatively regulates osteoblastogenesis and bone mass. Thus, identifying the mechanism by which BMP3 acts to depress bone formation may allow for the development of new therapeutics useful in the treatment for osteopenia and osteoporosis. Here, we report that cWnt signaling stimulates BMP3 expression in osteoblast (OB) lineage cells. The expression of BMP3 increases with OB differentiation. Treatment of cells with various cWnt proteins stimulated BMP3 expression. Mice with enhanced cWnt signaling had high expression levels of BMP3. Our data suggest that reduction in BMP3 levels may contribute beneficially to the positive effect of cWnt agonists on bone mass.

**Abbreviations**
ALP: alkaline phosphatase
BIO: bromoindirubin oxime
BMP: bone morphogenetic protein
BMSCs: bone marrow stromal cells
cWnts: canonical Wnts
DKK1: dickkopf‐related protein 1
ECR: evolutionarily conserved region
OBs: osteoblasts
OCYs: osteocytic cells
qPCR: quantitative real‐time PCR
sFRPs: secreted frizzled‐related proteins

Osteoporosis is a skeletal disease characterized by low bone mass and microarchitectural deterioration of bone tissue with a consequent increase in bone fragility and susceptibility to fracture 1. In 2010, more than 10 million Americans over the age of 50 had osteoporosis with another 43 million Americans at risk for the disease 2. It is estimated that greater than 1.5 million fragility fractures occur each year, with an annual healthcare cost of at least 14 billion US dollars 3. By 2025, the healthcare expenditures for osteoporotic fractures will approach 25.3 billion US dollars 4, highlighting the need for new therapies aimed at preventing the bone loss that normally occurs with aging 5.

Bone morphogenetic protein (BMP) and canonical Wnt (cWnt) signaling‐related molecules are known to regulate bone mass, fracture risk, fracture repair, and osteoblastogenesis. Generalized loss of endogenous BMP activity in postnatal mice through overexpression of BMP antagonists by osteoblast (OB) lineage cells leads to osteopenia, bone fragility, and spontaneous fracture 6, 7, 8. Mice lacking BMP2 are unable to maintain adequate bone formation after birth 9, while short‐term systemic administration of BMP2 has been reported to reverse bone loss in osteopenic mice 10.

Canonical Wnts are secreted proteins that signal through Lrp and Frizzled coreceptor complexes to stabilize intracellular pools of β‐catenin and activate Tcf/Lef‐dependent gene transcription 11. A large number of studies have examined the utility of enhancing cWnt signaling as means of promoting bone regeneration. LiCl treatment, through activation of cWnt signaling, improves fracture repair in mice, as does targeted inhibition of the Wnt pathway antagonists sFRP1, DKK1, or sclerostin 12, 13, 14.

BMP3 is the most abundant BMP within bone matrix and accounts for approximately 65% of the total BMP content in demineralized bone 15, 16. It is mainly secreted by OBs and osteocytes 17. Adult mice lacking BMP3 have increased bone mass, while mice with increased BMP3 levels in bone show delayed endochondral ossification with spontaneous rib fractures 18, 19. These phenotypes fit to BMP3 function; BMP3 suppresses OB differentiation by repressing BMP‐Smad signaling via interaction with activin receptor 2b (Acvr2b) *in vitro* 17.

Thus, identifying the mechanism by which BMP3 acts to depress bone formation may allow for the development of new therapeutics useful in the treatment for osteopenia and osteoporosis. However, as BMP3 is not a signaling molecule but acts as a receptor antagonist, we cannot identify downstream targets of BMP3 signaling. Instead, we need to focus on regulators that are upstream of BMP3 20. Here, we report that cWnt signaling is upstream of BMP3 and stimulates BMP3 expression in OB lineage cells.

## Materials and methods

### Collection of osteoblast lineage cells

Primary bone marrow stromal cells (BMSCs) were collected from femurs and tibias of 6‐week‐old wild‐type C57BL/6J mice or heterozygous BMP3 LacZ‐knock‐in reporter mice 17. Primary calvarial OBs were harvested by sequential collagenase digestion from natal wild‐type C57BL/6J mice, heterozygous BMP3 LacZ‐knock‐in reporter mice, or heterozygous DKK1‐knockout mice. Osteocytic cells (OCYs) were enriched by sequential collagenase digestion as reported previously 21, 22.

### Cell culture

Bone marrow stromal cells and OBs were treated with OB differentiation medium containing 50 μg·mL^−1^ ascorbic acid and 10 mm β‐glycerophosphate for 0, 7, or 14 days 23. BMSCs, OBs, OCYs, or 8‐week‐old male mice femoral bones were treated with 100 ng·mL^−1^ recombinant human BMP2 (R&D Systems, Minneapolis, MN, USA), several concentrations (0, 10, 20, 50, 100, or 200 ng·mL^−1^) of rhWnt3a (R&D Systems), 100 ng·mL^−1^ rhDkk1 (R&D Systems), 100 ng·mL^−1^ rhWnt1 (R&D Systems), 100 ng·mL^−1^ rhWnt5a (R&D Systems), 10 μm KCl (Sigma Aldrich Chemicals, St. Louis, MO, USA), or 10 μm LiCl (Sigma Aldrich Chemicals) for 1 day. HEK293T cells were maintained and cultured as reported previously 24.

### Administration of bromoindirubin oxime BIO and analysis of femoral bones from mice

Bromoindirubin oxime (BIO; Sigma Aldrich Chemicals) or control vehicle was administered intraperitoneally into 8‐week‐old wild‐type male mice (0.75 mg·kg^−1^ IP 3 times over 9) 25. Mice were sacrificed at 1 day after last BIO injection. Femoral bones were collected. Animal protocols were approved by the Harvard Medical Area Institutional Animal Care and Use Committee (Protocol #04043 to V.R.)

### Ectopic bone formation assay

The bone formation effects induced by BMP2 *in vivo* were examined using an ectopic bone formation assay 26. rhBMP2 (R& D Systems) (1 μg) were blotted onto a collagen sponge disk (6 mm diameter, 1 mm thickness) made from commercially available bovine collagen sheets (Helistat, Integra LifeSciences, Plainsboro, NJ, USA), freeze‐dried, and maintained at −20 until being implanted into the mice. All procedures were performed under sterile conditions. The mice were anesthetized using pentobarbital (Kyoritsu Seiyaku, Tokyo, Japan), and collagen pellets were surgically implanted into dorsal muscle pouches (2 pellets/animal) of the mice (8 weeks old).

### RNA isolation and quantitative real‐time PCR

Total RNA was isolated from cells using Trizol (Invitrogen, Carlsbad, CA, USA) and then reverse‐transcribed into cDNA using Transcriptor First Strand cDNA Synthesis Kit (Roche, Basel, Switzerland). The cDNA was amplified by quantitative real‐time PCR (qPCR) using primers specific for murine BMP3 (forward, tctcccaagtcatttgatgct; reverse, gcgtgatttgatggtttcaa), murine osteocalcin (OC) (forward, agactccggcgctacctt; reverse, ctcgtcacaagcagggttaag), murine SOST (forward, caggagaggaagcttgagtcc; reverse, agggtagaaagacccccatc), murine DKK1 (forward, ccgggaactactgcaaaaat; reverse, ccaaggttttcaatgatgctt), murine alkaline phosphatase (ALP) (forward, cggatcctgaccaaaaacc; reverse, tcatgatgtccgtggtcaat), and β‐actin (forward, aaggccaaccgtgaaaagat; reverse, gtggtacgaccagaggcatac). qPCR was performed in 96‐well plate using Fast Start Universal SYBR Green Master (Roche) with iCycler Multicolor Real‐Time PCR Detection system (BIO‐RAD, Richmond, CA, USA) 27. Values were normalized to β‐actin using the 2‐ΔΔ*C*_t_ method 28.

### β‐Galactosidase activity, cell transfection, and luciferase activity

β‐Galactosidase activity was determined by the Beta‐Glo Assay System (Promega, Madison, WI, USA) according to the manufacturer's instructions. HEK293T cells were transfected with plasmids using Lipofectamine 3000 (Invitrogen) according to the manufacturer's instruction. Luciferase assays were performed using pGL4.26‐ or pGL4.26‐containing R2 luciferase plasmid and phRL‐SV40 (Promega) with the Dual‐Glo Luciferase Assay System (Promega) as previously described 17.

### Chromatin immunoprecipitation assay

ChIP was performed with a ChIP assay kit (Cell signaling, Beverly, MA, USA) according to the manufacturer's instructions using anti‐β‐catenin rabbit polyclonal antibody (#9562; Cell Signaling) and normal rabbit IgG (MBL, Aichi, Japan). The purified DNA was analyzed by PCR using primers. The primer pairs for R1 (forward, TCA GTA TGT CTT GCT GGC GA; reverse, TTT TAT TAC CCG ACA CAG GTG), R2 (forward, TGT GAC TAT GGG TGA TGG AG; reverse, TTG CCA TTT GTT TAC TTT CTC C), or R3 (forward, GCT GCA AGG ACA TTT CAC AC; reverse, GAG AGG CTC CAA TGA GAT CA).

### Western blot analysis

The following antibodies were used for western blot analysis: anti‐BMP3 mouse monoclonal antibody (C‐9, sc390046; Santa Cruz, Santa Cruz, CA, USA), anti‐β‐catenin rabbit polyclonal antibody (#9562; Cell Signaling), and anti‐β‐actin mouse monoclonal antibody (Sigma Aldrich Chemicals).

### *In silico* experiments

DNA sequences were aligned using blastn 29 version 2.2.26± or ECR Brower 30 through the respective online servers or locally using muscle in mega5 software 31. The consensus sequence upstream of Bmp3 was constructed using the Los Alamos National Laboratory's Simple Consensus Maker (http://www.hiv.lanl.gov/content/sequence/CONSENSUS/consensus.html) using ‘Output aligned’ parameter. For the identification of transcription factor binding sites, DNA sequences were first aligned using zpicture 32.

### Plasmids

Sequences corresponding to specific regions upstream of murine Bmp3‐R2 [chr5:98846288–98846834 (547 bp)] were amplified and cloned into the promoter‐firefly luciferase reporter vector pGL4.26 (Promega). Mutant Bmp3‐R2 plasmid was generated using the following specific primer: 5′‐ctaaaatgctaattttggttttttttgagtcctgtgactatgggt‐3′ (mutation underlined). All of the final constructs were confirmed by sequencing.

### Statistical analysis

Comparisons were made on at least three independent experiments using an unpaired ANOVA with Tukey–Kramer *post hoc* test and Wilcoxon's signed rank test. The results are shown as the mean ± SD. The statistical significance is indicated as follows: **P < 0.01 and *P < 0.05.

## Results

### The expression of BMP3 increases with osteoblast differentiation

**Figure 1:**
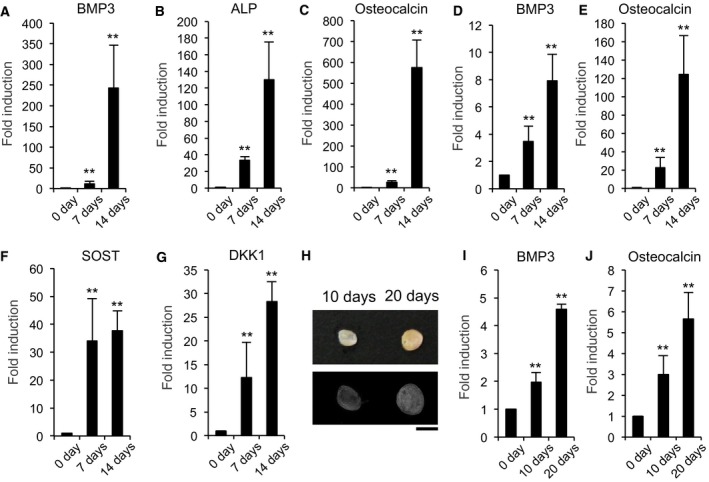
The expression of BMP3 increases with OB differentiation. BMSCs (A–C) and primary calvarial OBs (D–G) were treated with OB differentiation medium containing 50 μg·mL^−1^ ascorbic acid and 10 mm β‐glycerophosphate. The messenger RNA levels of BMP3 (A, D), ALP (B), OC (C, E), SOST (F), and DKK1 (G) were determined by qPCR on 0, 7, or 14 days (A–G). One microgram of BMP2 was implanted subfacially to induce ectopic bone formation in wild‐type mice (each time course *n* = 4). After 0, 10, or 20 days, the implants were removed and examined using soft X‐ray analysis. Represented pictures were shown. Scale bar corresponds to 5 mm (H). After 0, 10, or 20 days, the implants were determined for the expression levels of BMP3 (I) and OC (J) by qPCR. The data are expressed as the mean ± SD (*n* = 3). **P < 0.01, versus 0 day.

BMP3 is highly expressed by OBs and osteocytes, but very low in BMSCs and mesenchymal stem cells residing in the periosteum, which are progenitor cells for OBs and osteocytes 17. Thus, we monitored the expression levels of BMP3 in the process of OB differentiation and maturation *in vitro*. BMP3 expression in BMSCs and primary calvarial OBs increased during the process of OB differentiation and subsequent maturation (Fig. 1A,D). This increased BMP3 expression correlates with the expression levels of OB marker genes including ALP, OC, and the cWnt signal inhibitors such SOST (gene coding sclerostin) and DKK1 (Fig. 1B,C,E–G). BMP3 expression also increased during BMP2‐mediated ectopic bone formation in skeletal muscle tissue (Fig. 1H–J).

### cWnt signaling regulates BMP3 expression in osteoblast lineage cells

**Figure 2:**
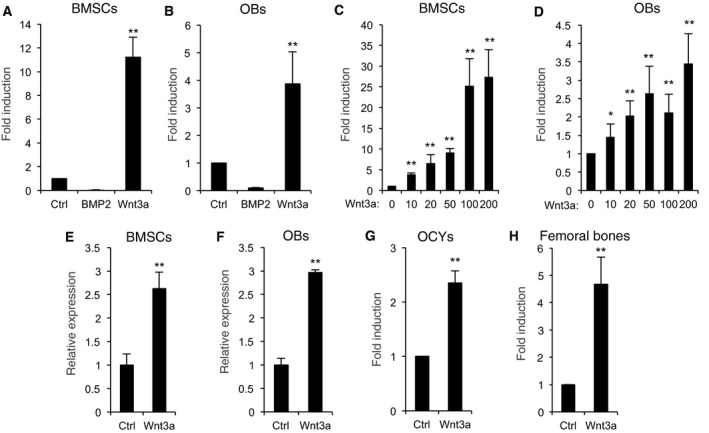
Wnt3a stimulates the expression levels of BMP3 in OB lineage cells. BMSCs (A) or OBs (B) from wild‐type mice were treated with control vehicle, 100 ng·mL^−1^ rhBMP2, or 100 ng·mL^−1^ rhWnt3a for 1 day. BMSCs (C) or OBs (D) were treated with 0, 10, 20, 50, 100, or 200 ng·mL^−1^ rhWnt3a. β‐Galactosidase activities were determined in BMSCs (E) and OBs (F) from heterozygous BMP3 LacZ‐knock‐in reporter mice on 1 day. OCYs (G) or femoral bones removed bone marrows (H) were cultured with or without 100 ng·mL^−1^ rhWnt3a. The messenger RNA levels of BMP3 were determined by qPCR on 1 day (A–D and G, H). The data are expressed as the mean ± SD (*n* = 3). **P < 0.01, *P < 0.05 versus control vehicle treatment (A–H).

Canonical Wnts and BMPs are produced by OB lineage cells and regulate osteoblastogenesis through several complex interactions 33. When we examined the effect of BMP2 or Wnt3a on BMP3 expression, we found that Wnt3a induced the mRNA levels of BMP3 in BMSCs and OBs, in a dose‐dependent manner, in contrast to BMP2 (Fig. 2A–D). Using BMSCs or OBs obtained from BMP3‐LacZ mice as a system to measure BMP3 levels, we found that Wnt3a greatly increases BMP3 production (Fig. 2E,F). When osteocytes and bone marrow flushed from femurs, which contained a large number of OBs and osteocytes, were treated with Wnt3a (Fig. 2G,H), the expression levels of BMP3 also increased, suggesting that Wnt3a regulates BMP3 expression in OB lineage cells.

**Figure 3:**
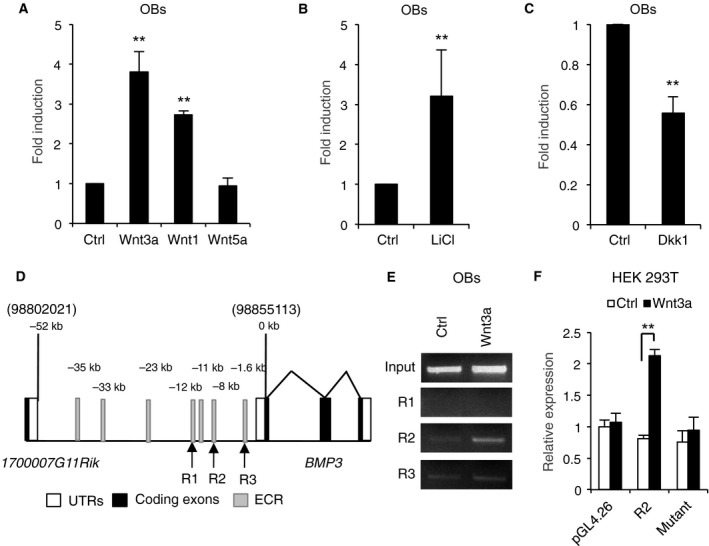
Canonical Wnt signaling regulates BMP3 expression. OBs were treated with control vehicle, 100 ng·mL^−1^ rhWnt3a, rhWnt1, or rhWnt5a (A), with 10 μm KCl (Ctrl) or LiCl (B), with or without 100 ng·mL^−1^ rhDKK1 for 1 day (C). The messenger RNA levels of BMP3 were determined by qPCR on 1 day (A–C). Schematics showed BMP3 gene and 5′ flanking region before 1700007G11Rik. Gray regions indicated ECR (humans and mouse) (≥ 77% homology); white regions and black regions indicated UTRs and coding regions of BMP3, respectively. R1, R2, and R3 indicated that the ECRs contained Tcf/Lef binding sites (D). OBs were treated with or without 100 ng·mL^−1^ rhWnt3a for 1 day. The chromatin from each sample was precipitated using anti‐β‐catenin antibody. The regions of R1, R2, or R3 were PCR‐amplified from the immunoprecipitated DNA (E). HEK293T cells were transfected with pGL4.26 backbone vector, R2 region‐containing pGL4.26 vector, or a R2 region‐containing pGL4.26 vector with mutant sequences of the Tcf/Lef binding site and then treated with or without 100 μg·mL^−1^ rhWnt3a (F). The data are expressed as the mean ± SD (*n* = 3). **P < 0.01 versus control (A–C, F).

Not only cWnt but also noncanonical Wnts are important for bone metabolism 34. We next examined whether other Wnts or the factors modulating Wnt signaling affect BMP3 expression. Wnt1, another cWnt, and with LiCl, an inhibitor of GSK3β also induced BMP3 expression, while treatment with Wnt5a, an activator of noncanonical Wnt signaling, had no effect on BMP3 expression. Furthermore, treatment of OBs with Dkk1, a potent cWnt signaling inhibitor, reduced basal levels of BMP3 (Fig. 3A–C). We previously reported the region from 0 to −0.8 kbp upstream of bmp3 in mammals containing promoter by comparative genomics and functional analyses 20. By same strategy, we used ECR Browser 32 to identify regions of nucleotide conservation between *Homo sapiens* (humans) and *Mus musculus* (mouse). We focused on murine chromosome 5 from position 98802021 to position 98855113, which corresponds to the entire region between the annotated murine BMP3 transcriptional start site and the nearby 1700007G11Rik open reading frame (Fig. 3D), because, in general, the cis‐regulatory regions reside at upstream of transcriptional start of the genes 20. This revealed that seven evolutionarily conserved regions (ECRs; ≥ 77% homology between humans and mouse) were identified and R1 (located at −12 kb), R2 (−8 kb), and R3 (−1.6 kb) contain putative Tcf/Lef binding sequences (Fig. 3D). ChIP analysis demonstrated that endogenous β‐catenin binds to R2 region when the cells were treated with Wnt3a (Fig. 3E). Furthermore, the luciferase reporter‐containing R2 region responded to Wnt3a and its activity increased (Fig. 3F), suggesting that at least in part cWnts–β catenin signaling directly regulates BMP3 expression via R2 region.

### Enhanced cWnt signaling has high expression levels of BMP3 in mice

**Figure 4:**
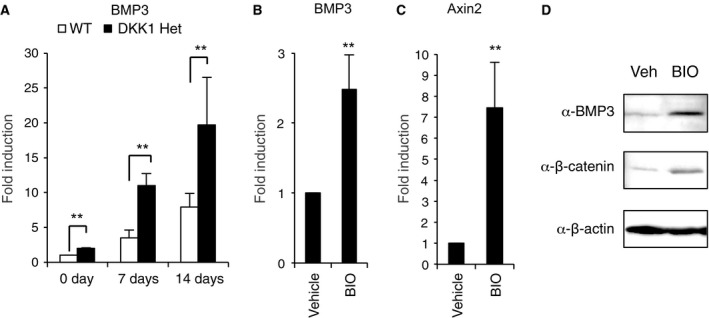
Enhanced cWnt signaling has high expression levels of BMP3 in the mice. OBs from heterozygous DKK1‐knockout mice or wild‐type littermates (WT) were treated with OB differentiation medium. The messenger RNA levels of BMP3 (A) were determined by qPCR on 0, 7, or 14 days (A). After injection of 0.75 mg·L^−1^ control vehicle or BIO on three times, the messenger RNA levels of BMP3 and Axin2 were determined by qPCR (A–C) and protein levels of BMP3, β‐catenin, and β‐actin were assessed by western blot analysis (D). The data are expressed as the mean ± SD (*n* = 3). **P < 0.01 versus wild‐type (A) or control vehicle treatment (B, C).

We finally examined whether the BMP3 expression increased in cWnt enhancing model *in vivo*. The expression levels of BMP3 were found to be increased in OBs from DKK1 heterozygous‐knockout mice, resulting in enhanced cWnt signaling and increased bone mass 35 (Fig. 4A). The inhibition of GSK3β by BIO injection increased mRNA levels of BMP3 and Axin2, which is direct target gene of cWnt signaling (Fig. 4B,C). Administration of BIO also stimulated the expression levels of β‐catenin and BMP3 in femoral bone *in vivo* (Fig. 4D).

## Discussion

In this report, we demonstrated that the BMP and cWnt signaling pathways interact at the level of BMP3. BMP signaling and cWnt signaling are essential for osteoblastogenesis, and crosstalk between both signaling is known. cWnt signaling stimulates the transactivation of BMP2 through the Tcf/Lef response elements in the BMP2 promoter 36. Activation of Wnt signaling also induces expression of BMP family members including BMP2, BMP4, and BMP7 and increases expression of BMP target genes such as Msx and gremlin in the mesenchyme 37. cWnt signaling also enhances BMPs expression in C3H10T1/2 cells 38, 39. In contrast, some *in vitro* studies demonstrated that BMPs induce cWnts in C2C12 cells and primary OBs 40, 41. However, BMP signaling upregulates sclerostin and DKK1 expression, leading to an inhibition of cWnt signaling and a decrease in bone mass 42, 43, 44. Thus, interpretation of crosstalk between BMPs and cWnts is sometimes controversial, and these physiological roles are still largely unknown. Our finding provides novel insights into the nature of functional crosstalk integrating the BMP and cWnt pathways in OB differentiation and skeletal homeostasis.

BMP3 is negative regulator of osteoblastogenesis and bone mass acting as acvr2b antagonist 17, 18, 45. Administration of Acvr2bFc, a second soluble type II Acvr2b decoy, also leads to an increased bone formation in adult mice 46. The importance of cWnt signaling in bone is well documented, and in general, increasing cWnt signaling correlates with enhanced bone formation through increased differentiation and maturation of OBs. More recently, neutralizing antibodies targeting antagonists of the cWnt pathway, such as SOST and DKK1, have entered clinical trials as systemic agents that enhance bone formation 11.

Taken together, the induction of BMP3 by cWnt seems to be negative feedback mechanism in the aspect of osteoblastogenesis and bone formation, suggesting that reducing BMP3 levels may provide an additional benefit to increasing bone mass using agents that enhance cWnt signaling. Needless to say, it is important to establish animal model and analysis. In conclusion, cWnt signaling upregulates BMP3 expression in OB lineage cells.

## Author contributions

SK performed the experiments. SK and VR reviewed the intermediate draft. SK and VR designed the study, performed the literature review, prepared the initial and final versions of the article, and submitted the document.
